# Protective effect of the total alkaloid extract from Bulbus *Fritillariae Pallidiflorae* on cigarette smoke-induced Beas-2B cell injury model and transcriptomic analysis

**DOI:** 10.29219/fnr.v68.10689

**Published:** 2024-06-11

**Authors:** Xiaoyu Wang, Xiao Liu, Er-Bu AGA, Wai Ming Tse, Kathy Wai Gaun Tse, Bengui Ye

**Affiliations:** 1 Key Laboratory of Drug-Targeting and Drug Delivery System of the Education Ministry, Sichuan Engineering Laboratory for Plant-Sourced Drug and Sichuan Research Center for Drug Precision Industrial Technology, West China School of Pharmacy, Sichuan University, Chengdu, Sichuan, China; 2 Medical College of Tibet University, Lasa, Tibet, China; 3 Nin Jiom Medicine Manufactory (H.K.) Limited, Hong Kong, China

**Keywords:** Bulbus Fritillariae Pallidiflorae, total alkaloid, COPD, cigarette smoke extract, Beas-2B cell, transcriptomic

## Abstract

**Background:**

Bulbus *Fritillariae Pallidiflorae* (BFP) is a traditional Chinese medicine that has long been used to treat lung diseases, but the active components and mechanism are still unclear.

**Objective:**

This study aimed to investigate the effect and mechanism of the total alkaloid extract from BFP (BFP-TA) on cigarette smoke extract (CSE)-induced Beas-2B cells injury.

**Design:**

The Beas-2B cells injury model was induced by 2% CSE, then the effect of BFP-TA on the levels of total antioxidant capacity (T-AOC), superoxide dismutase (SOD) and malondialdehyde (MDA) was detected according to the instructions of the T-AOC assay kit, the SOD detection kit and the MDA detection kit, and the production of ROS was detected by fluorescence microscopy. The effect of BFP-TA on Beas-2B cells apoptosis was detected by flow cytometry, and the effect of BFP-TA on related protein expression was detected by western blot. Subsequently, the effect of BFP-TA on differentially expressed genes (DEGs) in CSE-induced Beas-2B cells was studied by transcriptomic sequencing, and the expression of DEGs was verified by quantitative real-time polymerase chain reaction (qPCR).

**Results:**

The results showed that BFP-TA could attenuate CSE-induced oxidative damage in Beas-2B cells by elevating T-AOC and SOD levels while inhibiting ROS and MDA levels, and the mechanism was potentially related to the SIRT1/Nrf2/Keap1 signaling pathway. Furthermore, BFP-TA could inhibit CSE-induced apoptosis by inhibiting the protein expression of Bax, MST1 and FOXO3a, and exert anti-inflammatory effect by inhibiting the activation of MAPK signaling pathway. Subsequently, transcriptome analysis and qPCR validation showed that BFP-TA could alleviate inflammation, oxidative stress, apoptosis and lipid metabolism disorders by regulating the expression of DEGs in PPAR and PI3K-Akt signaling pathways, thereby exerting a protective effect against CSE-induced Beas-2B cell injury.

**Conclusion:**

This study is the first to demonstrate that BFP-TA could exert a protective effect on CSE-induced Beas-2B cell injury by exerting anti-inflammatory, antioxidant, anti-apoptotic and regulate lipid metabolism disorders.

## Popular scientific summary

- The total alkaloid extract from Bulbus *Fritillariae Pallidiflorae* as BFP-TA could attenuate cigarette smoke extract (CSE)-induced oxidative damage, apoptosis and inflammation in Beas-2B cells, and the mechanism may be related to the regulation of apoptosis-related proteins, SIRT1/Nrf2/Keap1 and MAPK signaling pathways.
- Transcriptomic analysis showed that BFP-TA may exert a protective effect on CSE-induced Beas-2B cells injury by regulating the expression of differentially expressed genes in PPAR and PI3K-Akt signaling pathways.

Chronic obstructive pulmonary disease (COPD) is a chronic progressive airway and lung disease characterized by persistent airflow limitation and not fully reversible (1). Typical clinical features of COPD include dyspnea, cough and sputum (2). Cigarette smoke is a major risk factor for COPD, which is a complex mixture of more than 6,000 compounds and more than 1,000 free radicals (3, 4). COPD currently affects more than 400 million people worldwide, with high prevalence and mortality rates, and the prevalence increases with age. According to the World Health Organization, COPD is predicted to be the third leading cause of death worldwide by 2030 (5). Current pharmacological treatments have limited impact on the progression or mortality of COPD, and there is little evidence that any one drug has the potential to reverse airway remodeling and obstructive airflow. Therefore, there is an urgent need to develop safe and effective new drugs for the treatment of COPD.

Bulbus *Fritillariae Pallidiflorae* (BFP) is the dried bulb of *Fritillaria walujewii* Regel or *Fritillaria pallidiflora* Schrenk from the Liliaceae family and was recorded in the Chinese Pharmacopoeia (2020 edition). BFP is mainly distributed in the northern Tianshan Mountains of Xinjiang around Yili, Tacheng, and Bortala (6), and is one of the famous local herbs in Xinjiang (7). Furthermore, BFP has the efficacy of moistening lungs, eliminating sputum, relieving cough, anti-inflammatory and antioxidant (8), which has not only been used to treat lung diseases for a long time but also widely used in the field of healthcare products and food (9). BFP contains various chemical components such as alkaloids, saponins, terpenoids, flavonoids, polysaccharides, coumarins, etc., among which isosteroid alkaloids are the main active components (10). The previous study of our research group showed that the isosteroid alkaloids (verticinone, verticine, imperialine-3-β-D-glucoside, delavine, imperialine, and peimisine) in *Fritillaria* have anti-inflammatory and antioxidant activities (11, 12). However, the pharmacological effect and biological activity of the total alkaloid extract from Bulbus *Fritillariae Pallidiflorae* (BFP-TA) have been less reported. Only a few articles have reported the pharmacological effects of BFP-TA: BFP-TA could attenuate airway remodeling through the Wnt/β-catenin signaling pathway in asthma model rats (13), and BFP-TA could exert antitussive and asthma-relieving effects on the ovalbumin-induced cough model guinea pigs (14). Furthermore, our research group has found that BFP-TA could improve lung function, attenuate the histopathology changes of airway and lung tissues, and inhibit the inflammatory response and oxidative stress in cigarette smoke-induced COPD mice (unpublished observation). However, the anti-COPD mechanism of BFP-TA is still unclear and needs further study.

Studies have shown that cigarette smoke extract (CSE) exposure induces persistent inflammation, oxidative stress, and apoptosis in human bronchial epithelial cells (15, 16). The airway epithelium is the first defense barrier against toxic particles, gases and pathogens. Therefore, in this study, CSE-induced human bronchial epithelial cells (Beas-2B cells) were used to mimic the COPD microenvironment *in vitro* to investigate the effect of BFP-TA on CSE-induced apoptosis, oxidative stress and inflammatory responses. Subsequently, the transcriptome analysis was used to further explore the molecular mechanism of BFP-TA in protecting against CSE-induced Beas-2B cells injury. This study aimed to provide data support for further investigating the anti-COPD effect and molecular mechanisms of BFP-TA and the development of new drugs for the treatment of COPD.

## Materials and methods

### Chemicals and reagents

Jiaozi cigarettes were purchased from Sichuan Zhongyan (Chengdu, China). Dexamethasone (Dexa, purity > 98%), 0.25% trypsin, and phosphatase inhibitor were purchased from Solaribo Life Sciences (Beijing, China). Radio immunoprecipitation assay (RIPA) buffer, reactive oxygen species (ROS) assay kit, total superoxide dismutase (SOD) detection kit, malondialdehyde (MDA) detection kit, and bicinchoninic acid (BCA) protein kit were purchased from Beyotime Biotechnology (Shanghai, China). Annexin V-FITC/PI Apoptosis Kit was purchased from APExBIO (Houston, USA). Total antioxidant capacity (T-AOC) assay kit was purchased from Jiancheng Bioengineering Institute (Nanjing, China).

The rabbit primary antibodies for p38 MAPK, p44/42 (Erk1/2), JNK, Bax, Nrf2, Keap1, and anti-rabbit horseradish peroxidase (HRP)-conjugated secondary antibodies were purchased from Affinity Biosciences (OH, USA). The rabbit primary antibodies for SIRT1, MST1, FOXO3a, p-p38 MAPK, p-p44/42 (p-Erk1/2), p-JNK, and β-actin were purchased from Cell Signaling Technology (Boston, USA).

### Preparation and material basis analysis of BFP-TA

BFP (purchased from the Gongliu County of Xinjiang Uygur Autonomous Region) are the dried bulbs of *Fritillaria pallidiflora* Schrenk from the Liliaceae family. The plant samples were identified by Professor Bengui Ye of Sichuan University, and the voucher specimens were deposited in the herbarium of the West China School of Pharmacy, Sichuan University. Based on the previous research of our research group (17, 18), BFP-TA was prepared by purification with a macroporous adsorbent resin. The content of total alkaloids was calculated with the method of ‘content determination’ in the ‘*Fritillariae Cirrhosae* Bulbus’ according to the Chinese Pharmacopoeia (2020 Edition). Subsequently, the material basis of BFP-TA was analyzed by HPLC-ELSD and UHPLC-MS/MS. The specific methods are described in the Supplementary Material.

### Cell culture

Beas-2B cells were purchased from ATCC (VA, UAS) and cultured in DMEM medium (Gibco, NY, USA), supplemented with 10% FBS (Gibco, NY, USA), and at 37°C with 5% CO_2_. Cells were grown to about 80% confluence in a 1:3 ratio of passaged culture, and the cells with good growth state and logarithmic growth stage were selected for follow-up experiments.

### Preparation of CSE

One Jiaozi cigarette was continuously smoked with a 50 mL syringe, and all the smoke was injected into 10 mL DMEM medium. After thorough shaking and mixing, the pH of the CSE solution was adjusted to 7.4, filtered with a sterile 0.22 μm needle filter, and the concentration of the CSE solution was set to 100% and used within 30 min.

### Cell viability assay

Cells in the logarithmic growth phase were inoculated in 96-well plates at a density of 5×10^3^ cells/well. After 24 h of incubation, different concentrations of CSE solution (0, 0.5, 1, 2, 5, 10, and 20%) were added to each well. After incubation for 24 h, 10 μL CCK-8 (APExBIO, Houston, USA) was added to each well and incubated for 4 h. The absorbance was then measured at 450 nm using a microplate reader (Bio-rad, Richmond, USA). The effect of different concentrations of BFP-TA (0, 1, 5, 10, 20, 40, 80, 160, 320 μg/mL) on the viability of Beas-2B cells was determined by the same method, and the cell viability (%) = (Abs_450_ of treated cells / Abs_450_ of control cells) × 100.

### Determination of T-AOC, SOD and MDA levels

Beas-2B cells in the logarithmic growth phase were inoculated in 6-well plates at a density of 2×10^5^ cells/well and then incubated for 24 h. Subsequently, the corresponding concentrations of CSE (0, 0.5, 1, 2, and 5%) were added to incubate with Beas-2B cells for 24 h. Then, the T-AOC level of the cells in each group was detected according to the instructions of the T-AOC assay kit, which indicated the selection of the concentrations of CSE. In the same way, the corresponding concentrations of BFP-TA (1, 5, 10, 20, 40 μg/mL) were added to incubate with 2% CSE-induced Beas-2B cells for 24 h. Next, the T-AOC level of the cells in each group was detected, which indicated the selection of the concentrations of BFP-TA. Subsequently, the levels of MDA and SOD in the cells of each group were detected according to the manufacturer’s instructions after the following grouping and treatments: the control group was added equal amount of DMEM medium; the CSE group was added 2% CSE; the Dexa group was added 2% CSE and 1 μM dexamethasone; the BFP-TA treatment groups were added 2% CSE and different concentrations of BFP-TA (5, 10, 20 μg/mL); then the cells were incubated for 24 h. If not otherwise specified, subsequent experiments were grouped and treated in the same way.

### ROS detection

Beas-2B cells were inoculated in 6-well plates at a density of 2×10^5^ cells/well and incubated for 24 h. Then 2% CSE and/or BFP-TA (5, 10, 20 μg/mL) were added to the CSE group and BFP-TA treatment groups, and the control group and positive control group were added equal amount of DMEM medium. After incubation for 24 h, the positive control solution (Rosup, 1:1000) was added to the positive control group and incubated at 37°C for 30 min. Then the culture medium of all groups was removed, and the cells were incubated for 20 min at 37°C in the dark after 1 mL diluted DCFH-DA (10 μM, diluted in DMEM medium) was added. Subsequently, the cells were washed three times with DMEM medium, and the ROS in each group of cells were detected by fluorescence microscope (Nikon, Tokyo, Japan) using an excitation wavelength of 488 nm and an emission wavelength of 525 nm.

### Apoptosis detection

Beas-2B cells were inoculated in 6-well plates at a density of 2 × 10^5^ cells/well. After 24 h of incubation, the cells were incubated with 2% CSE and BFP-TA (5, 10, 20 μg/mL) for 24 h. Then the medium was removed, the cells were digested with EDTA-free trypsin, and the cell suspension was collected and centrifuged (1,000 rpm, 3 min). Subsequently, the cells were resuspended in 500 μL 1×Binding Buffer, and 5 μL Annexin V-FITC and 5 μL Propidium lodide were added. After incubating away from light for 10 min, the apoptosis rate of each group was detected by flow cytometry (Agilent, CA, USA).

### Western Blot

Total proteins from Beas-2B cells were extracted using RIPA lysis buffer (RIPA: PMSF: phosphatase inhibitor = 100:1:1), and the protein content in each group was quantified with the BCA protein assay kit. After adding the sample loading buffer, the protein samples were heated at 100°C for 10 min. Equal amounts of proteins were separated by sodium dodecyl sulfate-polyacrylamide gel electrophoresis (SDS-PAGE) and transferred to polyvinylidene fluoride membranes (Millipore, MA, USA). Subsequently, the membrane was blocked with 5% skim milk for 1 h at room temperature and incubated with specific primary antibodies (1:1000) at 4°C overnight. After washing with tris buffered saline with tween 20 (TBST), the membrane was incubated with anti-rabbit HRP-conjugated secondary antibodies (1:10000) for 1 h at room temperature. Afterward, the membrane was washed with TBST, and protein bands were detected on the GelView 5000Plus Gel Imaging System (Biolight, Guangzhou, China) using the BeyoECL Plus (Millipore, MA, USA) and analyzed with ImageJ software.

### Transcriptome Sequencing

Beas-2B cells were divided into the control group (C), 2% CSE-induced group (CSE), 2% CSE + 20 μg/mL BFP-TA-treated group (BFP), with three biological replicates in each group. The total RNA was prepared based on the instructions of TRIzol reagent (Thermo, MA, USA), then mRNA was separated from the total RNA, and the mRNA was cleaved into small fragments, which were separated at 300 bp length. Subsequently, mRNA was synthesized into cDNA with reverse transcriptase, and the library was constructed, and sequenced by the Illumina Novaseq 6000 platform. The RNA isolation, quantitative library preparation and RNA sequencing services were provided by OE Biotech, Inc. (Shanghai, China).

### Bioinformatic analysis

Fastp (version 0.20.1), RseQC (version 4.0.0) and fastpc (version 0.11.9) software were used for quality control of sequencing data; hisat2 (version 2.1.0) software was used for genome comparisons; samtools (version 1.9) software was used for sam and bam file analysis; htseq-count (version 0.11.2) software was used for gene quantification; and DESeq2 (version 1.22.2) software was used for biological replicates and pairwise difference analysis. The significantly differentially expressed genes (DEGs) were screened according to the parameters |Fold Change| > 2 (|log_2_FC| > 1) and q < 0.05. The Gene Ontology (GO) database and the Kyoto Encyclopedia of Genes and Genomes (KEGG) database were used for the gene function and pathway enrichment analysis of DEGs.

### Quantitative real-time PCR

Total RNA from Beas-2B cells was extracted using TRIzol reagent. The total RNA in each sample was quantified by NanoDrop 2000 Spectrophotometers (Thermo Scientific, Waltham, USA). The cDNA was obtained by reverse transcription from RNA samples with the cDNA Reverse Transcription Kit (Takara, Beijing, China). Subsequently, quantitative real-time PCR (qPCR) assays were performed using SYBRPIME qPCR set (Baoguang Bio., Chongqing, China) on Bio-Rad CFX96 Real-Time System (Bio-Rad, Hercules, USA). The 2^−∆∆Ct^ method was used to calculate the relative gene expression, and GAPDH was the reference gene. GAPDH (No. B662104) and other primers were purchased from Sangon Biotech (Shanghai, China), and primer sequences are shown in Table 1.

**Table 1:** Primers for qPCR

Gene	Forward primer	Reverse primer
CCND1	5’-ATCAAGTGTGACCCGGACTG-3’	5’-CTTGGGGTCCATGTTCTGCT-3’
EPHA2	5’-ATTAAGGACTCGGGGCAGGA-3’	5’-CTGCATCAGGTCCCACTTCC-3’
DDIT4	5’-TTAGCAGTTCTCGCTGACCG-3’	5’-GGTAAGCCGTGTCTTCCTCC-3’
ITGB4	5’-GAGGTAGGTCCAGGACGGG-3’	5’-GGCTCCTTCAGCTTCTCAGG-3’
MMP1	5’-AGAGCAGATGTGGACCATGC-3’	5’-TTGTCCCGATGATCTCCCCT-3’
ANGPTL4	5’-CCTCTCCGTACCCTTCTCCA-3’	5’-AAACCACCAGCCTCCAGAGA-3’
FADS2	5’-ACCCTTTGTTTGTGTGTGCG-3’	5’-CGCGGAAGGCATCCTGTT-3’
SCD	5’-TTCCCGACGTGGCTTTTTCT-3’	5’-AGCCAGGTTTGTAGTACCTCC-3’
OLR1	5’-CCGGCAACAAGCAGAAGAAG-3’	5’-TGCTGGATGAAGTCCTGAACA-3’

### Statistical analysis

The data were expressed as mean ± SD, multiple comparisons were performed using One-way ANOVA followed by the Bonferroni post-test. All analyses were performed using GraphPad Prism 8 (San Diego, CA, USA). The value of *P* < 0.05 was considered statistically significant.

## Results

### Material basis research of BFP-TA

**Fig. 1:**
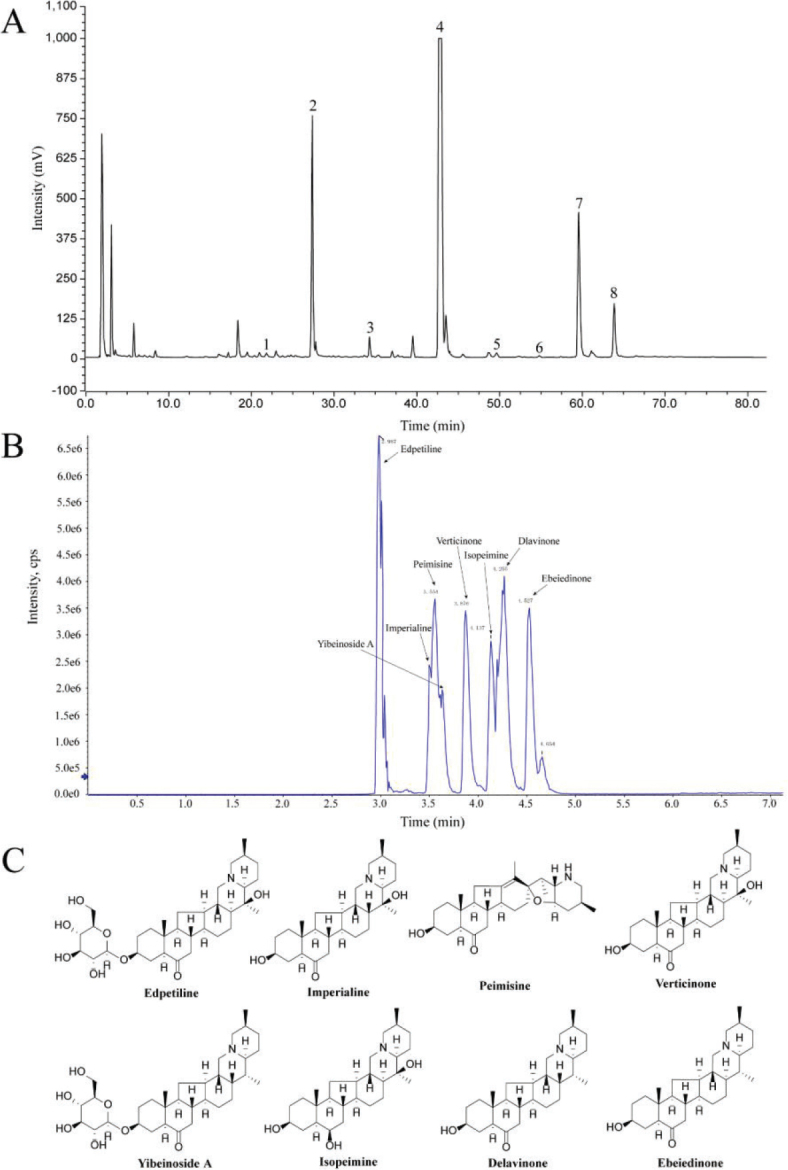
Material basis research of BFP-TA. (A) HPLC diagram of BFP-TA (B) The total ion chromatogram of eight alkaloids in a mixed standard solution by UHPLC-MS/MS. (C) Chemical structures of eight alkaloids.

According to the linear relationship between alkaloid concentration and OD415 (*Y* = 0.9708*X* − 0.0004, *R*^2^ = 0.9994), the total alkaloid content in BFP-TA is 64.28%. Subsequently, the BFP-TA was analyzed in HPLC-ELSD chromatographic conditions, and eight alkaloids in BFP-TA were identified by comparing the retention time with reference compounds, which is shown in Table 2 and Fig. 1A. UHPLC-MS/MS was used to determine the content of eight alkaloids in BFP-TA and the results are shown in Table 2. The total ion chromatogram of eight alkaloids is shown in Fig. 1B, and the chemical structures of the eight alkaloids are shown in Fig. 1C. Furthermore, the linear relationships and ranges of the eight reference compounds are shown in Supplementary Table 1, and the mass spectrum detection data of eight alkaloids are shown in Supplementary Table 2.

**Table 2:** Material basis research of BFP-TA

No	Identification compound	Retention Time (min)	Content (%)
1	Peimisine	21.91	0.22
2	Edpetiline	27.45	13.29
3	Yibeinoside A	34.49	1.18
4	Imperialine	42.74	34.68
5	Verticinone	49.87	0.80
6	Isopeimine	55.01	0.24
7	Delavinone	59.63	6.92
8	Ebeiedinone	64.02	3.28

### Effect of CSE and BFP-TA on Beas-2B cell viability

**Fig. 2:**
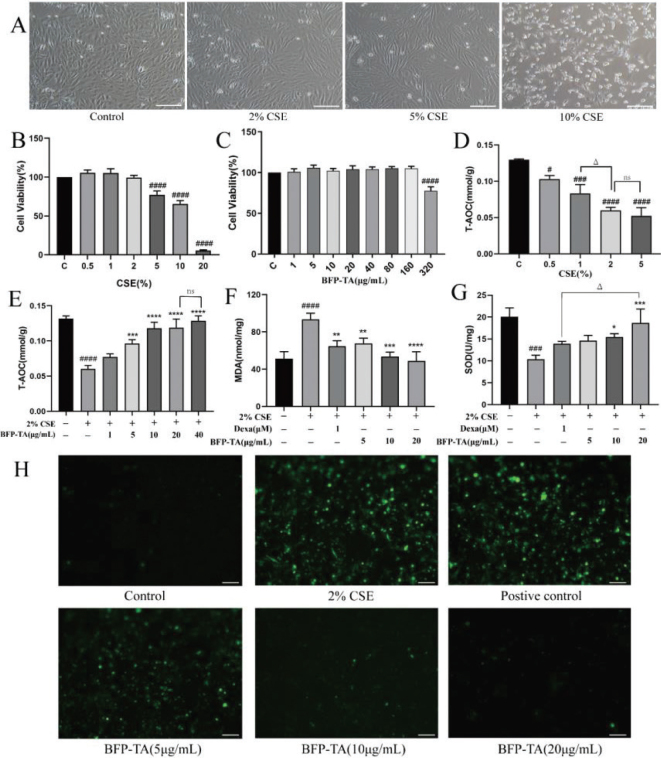
BFP-TA attenuates CSE-induced oxidative stress in Beas-2B cells. (A) Morphology of Beas-2B cells after treating with different concentrations of CSE for 24 h, scale 100 μm. (B, C) The viability of Beas-2B cell after treated with different concentrations of CSE or BFP-TA was determined by the CCK8 method. (D, E) The T-AOC level of Beas-2B cells after treating with different concentrations of CSE and/or BFP-TA was determined by the T-AOC assay kit. (F, G) Effect of BFP-TA on MAD and SOD levels in 2% CSE-induced Beas-2B cells. (H) The effect of BFP-TA on ROS production in 2% CSE-induced Beas-2B cells was detected by fluorescence microscopy, scale 100 μm. *n* = 3, ^#^*P* < 0.05, ^###^*P* < 0.001, ^####^*P* < 0.0001 compared with the control group; ^*^P < 0.05, ^**^P < 0.01, ^***^P < 0.001, ^****^P < 0.0001 compared with the CSE group.

As shown in Fig. 2A, the Beas-2B cells were pike-shaped, full-shape, and in good growth status within the concentration of 5% CSE. After 10% CSE treatment, the morphology of Beas-2B cells was markedly altered, with a large number of cells deaths and crumples. As shown in Fig. 2B, the viability of Beas-2B cells was significantly decreased with 5% and higher concentrations of CSE treatment (*P* < 0.0001). As shown in Fig. 2C, BFP-TA had no significant effect on cell viability less than 160 μg/mL. Based on these results, subsequent experiments will further determine the optimal administration concentration of CSE and BFP-TA within the safe dose range.

### Effect of CSE and BFP-TA on T-AOC level in Beas-2B cells

CSE induction leads to oxidative damage to intracellular lipids, proteins and DNA, and the level of intracellular T-AOC shows the total levels of various intracellular antioxidant molecules and enzymes. As shown in Fig. 2D, the level of T-AOC gradually decreased with the increase in CSE concentration, and 2% CSE-induced significantly reduced intracellular T-AOC (*P* < 0.0001). Considering the effect of different concentrations of CSE on cell viability, the Beas-2B cell injury model was induced by 2% CSE. As shown in Fig. 2E, the administration of 5, 10, 20 and 40 μg/mL of BFP-TA all significantly inhibited the decrease of T-AOC in 2% CSE-induced Beas-2B cells (*P* < 0.05, *P* < 0.001, *P* < 0.0001, *P* < 0.0001), and the effect was no significant difference between 20 and 40 μg/mL. Therefore, 5, 10, and 20 μg/mL BFP-TA were used for subsequent experiments.

### Effect of BFP-TA on MDA, SOD and ROS production in Beas-2B cells

As shown in Fig. 2F, G, 2% CSE induced a significantly increased in intracellular MDA level (*P* < 0.0001) and a significantly decreased in SOD level (*P* < 0.001). Compared with the CSE group, different doses of BFP-TA groups all significantly decreased the MDA level (*P* < 0.01, *P* < 0.001, *P* < 0.0001), and medium- and high-dose BFP-TA groups significantly increased the SOD level (*P* < 0.05, *P* < 0.001), among which high-dose BFP-TA group had a better effect than the dexamethasone group (*P* < 0.05). These results suggest that BFP-TA could attenuate CSE-induced oxidative damage in Beas-2B cells by decreasing the level of MDA and elevating the level of SOD. Cigarette smoke contains a large amount of oxidants and free radicals, which can stimulate intracellular ROS production and cause oxidative damage. As shown in Fig. 2H, 2% CSE induced a large amount of ROS production in Beas-2B cells, and different doses of BFP-TA administration all decreased intracellular ROS level, thereby protecting the cells from CSE-induced oxidative damage.

### Effect of BFP-TA on CSE-induced apoptosis in Beas-2B cells

**Fig. 3:**
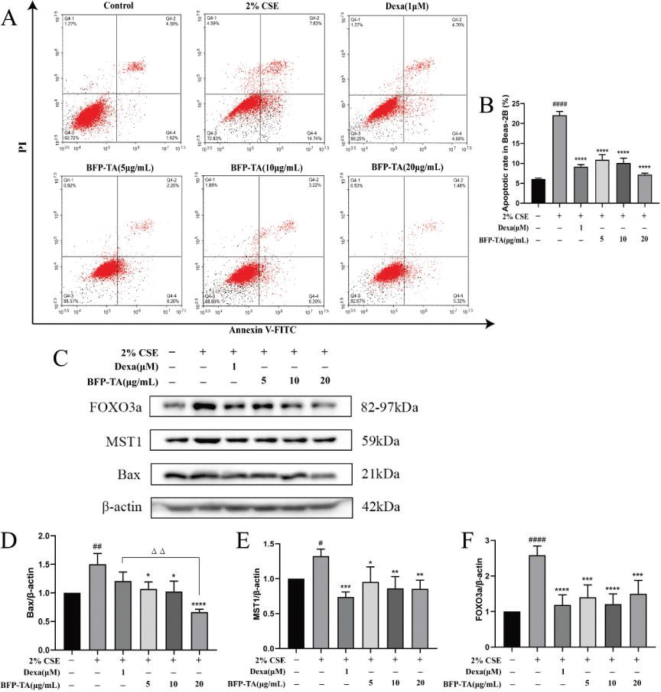
Effect of BFP-TA on CSE-induced apoptosis in Beas-2B cells. (A) After treating with different concentrations of BFP-TA or dexamethasone, the apoptosis in 2% CSE-induced Beas-2B cells was detected by flow cytometry. (B) Statistical analysis of apoptosis rate in each group. (C–F) The effect of BFP-TA on the proteins expression of FOXO3a, MST1 and Bax in 2%CSE induced Beas-2B cells were detected by western blot. *n* = 3, ^#^*P* < 0.05, ^##^*P* < 0.01, ^####^*P* < 0.0001 compared with the control group; ^*^P < 0.05, ^**^P < 0.01, ^***^P < 0.001, ^****^P < 0.0001 compared with the CSE group.

Lung epithelial cell apoptosis is an important factor in the pathogenesis of COPD. As shown in Fig. 3A, B, the apoptosis rate was significantly increased in 2% CSE-induced Beas-2B cells (*P* < 0.0001), and different doses of BFP-TA administration groups all significantly reduced the apoptosis rate (*P* < 0.0001). Meanwhile, dexamethasone administration (1 μM) also significantly reduced the apoptosis rate (*P* < 0.0001). These results suggest that BFP-TA has a protective effect against CSE-induced apoptosis, and the effect is similar to that of dexamethasone.

As shown in Fig. 3C–F, the expression of pro-apoptotic protein Bax was significantly increased in 2% CSE-induced Beas-2B cells (*P* < 0.01), and the protein expression of Bax could be significantly inhibited in different doses of BFP-TA administration groups (*P* < 0.05, *P* < 0.05, *P* < 0.0001). Mammalian Sterile 20-like kinase 1 (MST1) plays an important role in multiple stress-induced apoptosis. Studies have shown that MST1 will be activated by apoptotic stimuli, which in turn activate downstream targets JNK/p38 and Forkhead box O (FOXO) protein family (19, 20). In this study, the protein expression of MST1 and FOXO3a was significantly increased in 2% CSE-induced Beas-2B cells (*P* < 0.05, *P* < 0.0001), and different doses of BFP-TA administration groups could inhibit the expression of MST1 and FOXO3a. These results suggested that BFP-TA could attenuate CSE-induced apoptosis by inhibiting the proteins expression of Bax, MST1 and FOXO3a.

### Effect of BFP-TA on SIRT1/Nrf2/Keap1 protein expression in CSE-induced Beas-2B cells

**Fig. 4:**
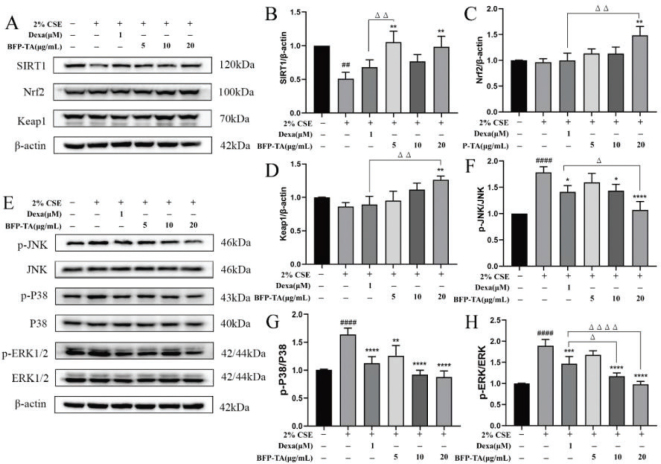
Effect of BFP-TA on protein expression in 2% CSE-induced Beas-2B cells was detected by western blot. (A–D) Effect of BFP-TA on SIRT1, Nrf2 and Keap1 protein expression in 2% CSE-induced Beas-2B cells. (E-H) Effect of BFP-TA on p-JNK, JNK, p-P38, P38, p-ERK1/2, and ERK1/2 protein expression in 2% CSE-induced Beas-2B cells. *n* = 3, ^##^*P* < 0.01, ^####^*P* < 0.0001 compared with the control group; ^*^P < 0.05, ^**^P < 0.01, ^***^P < 0.001, ^****^P < 0.0001 compared with the CSE group.

Silent information regulator 1 (SIRT1) plays an important role in the pathogenesis of COPD, which will be downregulated in response to oxidative stress stimuli. Nuclear factor erythroid 2-related factor 2 (Nrf2) and Kelch-like ECH-associated protein 1 (Keap1) are the main intracellular antioxidant defense systems. As shown in Fig. 4A–D, the expression of SIRT1 was significantly reduced in 2% CSE-induced Beas-2B cells (*P* < 0.01). Compared with the CSE group, low- and high-dose BFP-TA significantly inhibited the decrease of SIRT1 protein (*P* < 0.01), and high-dose BFP-TA significantly elevated the expression of Nrf2 and Keap1 proteins (*P* < 0.01). These results suggest that BFP-TA could attenuate CSE-induced oxidative damage by regulating the SIRT1/Nrf2/Keap1 signaling pathway.

### Effect of BFP-TA on MAPK signaling pathway in CSE-induced Beas-2B cells

As shown in Fig. 4E–H, compared with the control group, the levels of p-JNK/JNK, p-P38/P38, and p-ERK/ERK were significantly increased in 2% CSE-induced Beas-2B cells (*P* < 0.0001). The expression of p-JNK/JNK was significantly inhibited in medium- and high-dose BFP-TA groups (*P* < 0.05, *P* < 0.0001), among which the effect of the high-dose BFP-TA group was better than dexamethasone group (*P* < 0.05). Furthermore, the expression of p-P38/P38 was significantly inhibited in different doses of BFP-TA (*P* < 0.01, *P* < 0.0001, *P* < 0.0001). The expression of p-ERK/ERK was significantly inhibited in the medium- and high-dose BFP-TA groups (*P* < 0.0001), and the effect was better than dexamethasone group (*P* < 0.05, *P* < 0.0001). These results suggest that BFP-TA could reduce the CSE-induced inflammatory response in Beas-2B cells by inhibiting the activation of the MAPK signaling pathway.

### Sequencing data quality control and inter-sample correlation analysis

**Fig. 5:**
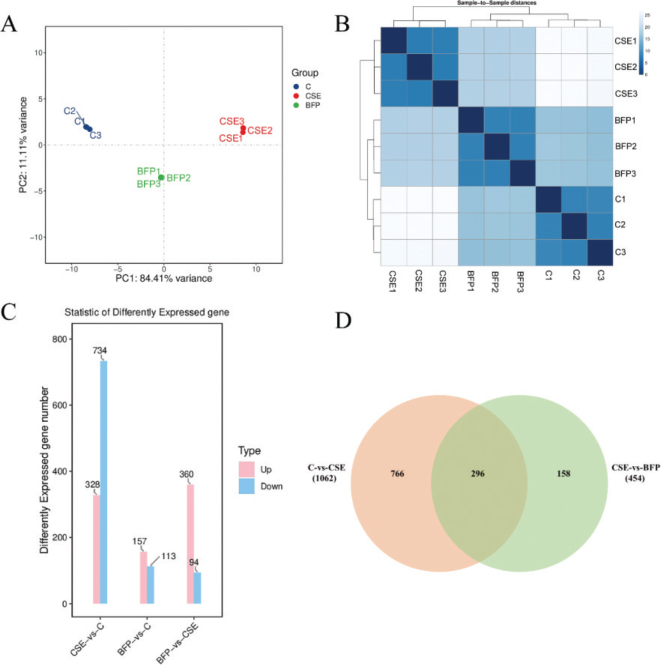
The inter-sample correlation analysis and DEGs number analysis. (A) Inter-sample principal component analysis. (B) Inter-sample cluster analysis. (C) Histogram of DEGs number between groups. (D) Venn diagram of DEGs number between groups.

Subsequently, transcriptomic analysis was applied to further investigate the mechanism of BFP-TA protects against CSE-induced Beas-2B cell injury. This study completed the transcriptome sequencing of 9 samples, and the quality control results of the sequencing data for each sample are shown in Table 3. Then, correlation analyses of gene expression levels between samples were performed to test the reliability of sample selection and experimental procedures and assess the biological reproducibility of samples within groups and the otherness between groups (Fig. 5A, B). The results showed that the similarity of the samples within each group was high, indicating that this experiment had good intra-group reproducibility. However, the difference between group C and group CSE was larger, which could be reduced after the intervention of the BFP.

**Table 3:** Quality control data of transcriptome

Sample	Clean reads (M)	Clean bases (G)	Valid Bases (%)	Q30 (%)	GC content (%)
C1	49.10	6.99	93.23	92.77	49.47
C2	49.24	6.93	91.99	93.26	49.49
C3	48.83	6.89	92.14	92.95	49.44
CSE1	49.49	6.97	92.04	93.56	49.57
CSE2	49.84	7.01	91.90	93.14	49.50
CSE3	50.05	7.03	91.79	93.31	49.71
BFP1	49.47	6.91	91.34	93.40	49.70
BFP2	49.09	6.89	91.59	92.86	49.23
BFP3	50.51	7.06	90.96	92.78	49.41

### The screening of DEGs

Based on the expression levels of genes in different samples, the DEGs were screened according to the parameters |log_2_FC| > 1 and *q* < 0.05. There were 3 different subgroups in this study, and the number of DEGs between each group is shown in Fig. 5C. Compared with the Control group, 328 DEGs were upregulated and 734 DEGs were downregulated in the CSE group. After treating with BFP-TA, 360 DEGs were upregulated and 94 DEGs were downregulated compared to the CSE group. As shown in Fig. 5D, there were 1062 DEGs in the Control group compared to the CSE group, and 454 DEGs in the CSE group compared to the BFP group. Furthermore, the intersection of ‘C vs. CSE’ and ‘CSE vs. BFP’ was 296 DEGs, which were closely related to COPD and significantly reversed after BFP treatment. Therefore, this study considers the 296 DEGs to be the key DEGs for the anti-COPD effect of BFP-TA.

### Transcriptome expression analysis of DEGs

**Fig. 6:**
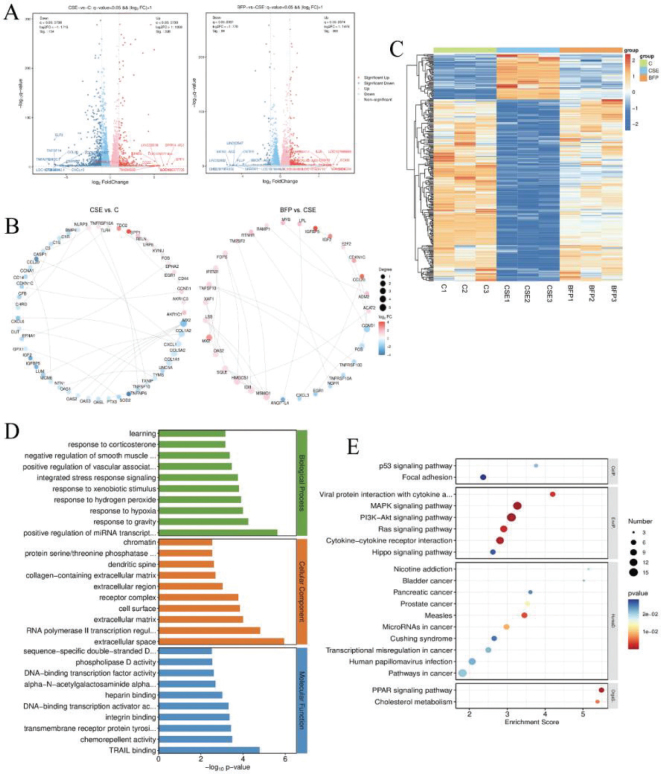
Transcriptomic analysis of 2% CSE-induced Beas-2B cells after treating with BFP-TA. (A) Volcano plots of DEGs. (B) PPI interaction diagram of top 30 DEGs. (C) Heatmap of key DEGs. (D) GO analysis of key DEGs. (E) KEGG analysis of key DEGs.

As shown in Fig. 6A, the volcano plot of DEGs was constructed to quickly assess the distribution of DEGs, and the Top 10 genes names of up- and down-regulation were labeled in the plot. The interrelationships of genes were obtained based on the annotation of species information in the STRING database, and the PPI interrelationship map was drawn based on the DEGs ranked in the top 30 (Fig. 6B). Subsequently, the cluster analysis was performed based on the screened key DEGs (Fig. 6C). The horizontal coordinate is the group, and the vertical coordinate is the DEGs, and the genes with similar clustered expression patterns may have similar functions or participate in the same biological process. The results showed that 237 genes were upregulated and 59 genes were downregulated after administration compared with the CSE group.

### GO and KEGG enrichment analysis of DEGs

Subsequently, the GO enrichment analysis was performed based on the screened key DEGs, and the gene functions of the DEGs were described based on the results of GO annotation, including three major categories: biological process (BP), cellular component (CC), and molecular function (MF). As shown in Fig. 6D, the GO enrichment analysis of the top 30 (filter the GO entries corresponding to PopHits ≥ 5 in the three categories and sort from largest to smallest according to the -log_10_ *P*-value) was found to be mainly related to response to hypoxia, response to xenobiotic stimulus, integrated stress response signaling, extracellular matrix, etc.

The KEGG database was used to analyze the pathway enrichment of key DEGs based on the KEGG annotation results, and the enrichment significance of DEGs in each pathway was calculated using the hypergeometric distribution test. The results include four major categories: cellular processes (CellP), environmental information processing (EnvIP), human diseases (HumaD), and organismal systems (OrgaS). As shown in Fig. 6E, the KEGG enrichment analysis of the top 20 (filter the pathway entries corresponding to PopHits ≥ 5, and sort from largest to smallest according to the -log_10_ *P*-value) was mainly enriched in p53 signaling pathway, PI3K-Akt signaling pathway, MAPK signaling pathway, PPAR signaling pathway, nicotine addiction, etc. The DEGs contained in the Top 20 signaling pathways of the KEGG enrichment analysis are shown in Supplementary Table 3.

### The expressions of DEGs were verified by qPCR

**Fig. 7:**
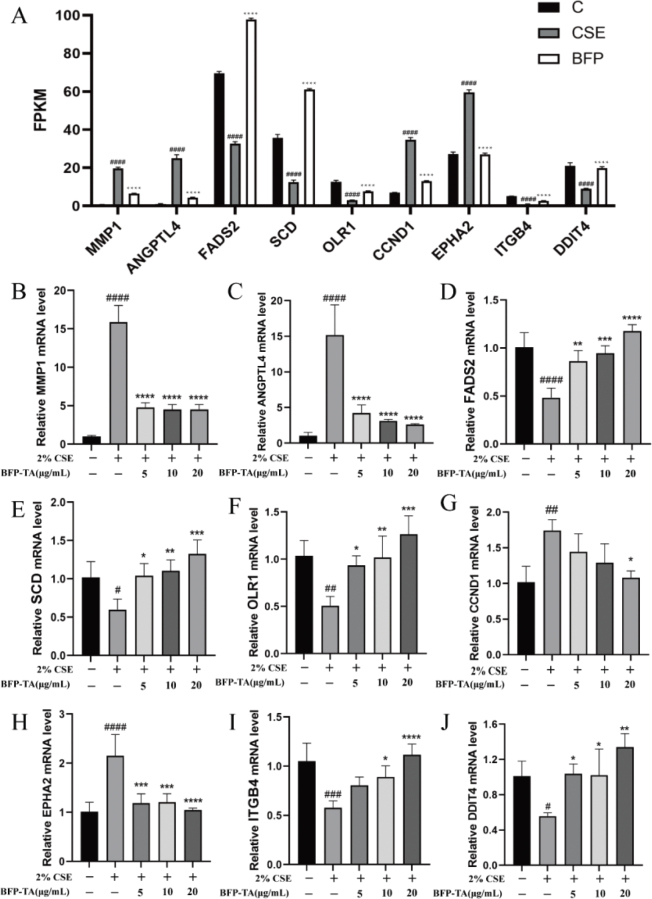
The effect of BFP-TA on the gene expression levels of Beas-2B cells. (A) The genes expression levels of DEGs in each group of cells. (B–J) The mRNA expression levels of MMP1, ANGPTL4, FADS2, SCD, OLR1, CCND1, EPHA2, ITGB4 and DDIT4 in each group of cells were verified by qPCR, and GAPDH was the reference gene. *n* = 3, ^#^*P* < 0.05, ^##^*P* < 0.01, ^###^*P* < 0.001, ^####^*P* < 0.0001 compared with the control group; ^*^P < 0.05, ^**^P < 0.01, ^***^P < 0.001, ^****^P < 0.0001 compared with the CSE group.

Based on the results of KEGG pathway analysis, DEGs contained in pathways related to inflammatory response, apoptosis, oxidative stress, and lipid metabolism, including the PI3K-AKT signaling pathway (16 DEGs), MAPK signaling pathway (14 DEGs) and the PPAR signaling pathway (6 DEGs). As shown in Fig. 7A, compared with the control group, the gene expression levels of FADS2, SCD, OLR1, ITGB4, and DDIT4 were significantly decreased (*P* < 0.0001), while MMP1, ANGPTL4, CCND1, and EPHA2 were significantly increased in 2% CSE-induced Beas-2B cells (*P* < 0.0001). The specific expression levels of DEGs are shown in Supplementary Table 4.

Subsequently, the expression of DEGs was verified by qPCR. As shown in Fig. 7B–J, the mRNA expression levels of MMP1, ANGPTL4, CCND1 and EPHA2 were significantly increased in CSE-induced Beas-2B cells (*P* < 0.0001, *P* < 0.0001, *P* < 0.01, *P* < 0.0001), while the mRNA expression levels of FADS2, SCD, OLR1, ITGB4, and DDIT4 were significantly decreased (*P* < 0.0001, *P* < 0.05, *P* < 0.01, *P* < 0.001, *P* < 0.05). And the BFP-TA administration reversed the above trends. Overall, the qPCR results were consistent with those of transcriptome sequencing, suggesting the reliability of transcriptome sequencing.

## Discussion

This study aimed to investigate the protective effect and molecular mechanism of BFP-TA in CSE-induced Beas-2B cell injury model, and to provide preliminary data support for demonstrating the protective effect of BFP-TA in the disease process of COPD. COPD is usually caused by regular exposure to smoking, toxic gases, dust particles, air pollution, and systemic inflammation of the lungs, etc., among which smoking is recognized as the most important factor contributing to COPD (2). Studies have shown that cigarette smoke can enter the lungs through the airways and directly damage bronchial and alveolar epithelial cells (3, 4). Therefore, 2% CSE-induced Beas-2B cell injury model was used to mimic the pathological microenvironment of COPD *in vitro* in this study. Furthermore, the pathogenesis of COPD is very complex and currently recognized mechanisms include chronic inflammation, oxidative stress, protease/antiprotease imbalance, airway remodeling, apoptosis, etc. (21–23). Therefore, in this study, we first investigated the effects of BFP-TA on oxidative stress, apoptosis, and inflammatory responses in CSE-induced Beas-2B cells.

As is well known, smoking-induced oxidative stress is an important factor in the development of COPD (24), and ROS are important signaling molecules in redox homeostasis and cellular damage. MDA is the end product of lipid peroxidation, and SOD is an important antioxidant enzyme in the organism. When the organism is stimulated by smoke, ROS and MDA will be largely produced, destroying the antioxidant defense system and causing damage to lung tissue (25). In this study, 2% CSE-induced oxidative stress marker levels (MDA and ROS) increased while T-AOC and SOD levels decreased, indicating that the cells were damaged by oxidative stress, while BFP-TA could alleviate the effect of CSE, thereby reducing intracellular oxidative damage. Furthermore, SIRT1 is a member of the NAD-dependent deacetylase family, which plays important roles in biological processes such as oxidative stress, inflammatory responses and apoptosis (26). Under normal physiological conditions, Nrf2 binds to Keap1 in the cytoplasm and is maintained at low level by Keap1-mediated ubiquitination and 26S proteasome-mediated degradation. When the body is stimulated by oxidative stress, Nrf2 will dissociate from Keap1, translocate to the nucleus, and bind with antioxidant response elements to promote the transcription of a variety of cellular defense molecules (27). In this study, western blot results showed that BFP-TA could increase the protein levels of SIRT1, Nrf2 and Keap1 in CSE-induced Beas-2B cells, suggesting that BFP-TA may attenuate the oxidative damage in CSE-induced Beas-2B cells by regulating the STRT1/Nrf2/Keap1 signaling pathway.

Previous studies have shown that CSE can induce apoptosis in human bronchial epithelial cells (HBECs) with a significant increase in the expression of pro-apoptotic protein Bax, which was consistent with this study (28). MST1 plays a key role in pro-apoptotic signaling, which is activated by apoptotic stimuli, and in turn activates downstream targets (JNK/p38, histone H2B and FOXO) and exerts pro-apoptotic functions (20, 29). In this study, we found that BFP-TA could inhibit CSE-induced apoptosis in Beas-2B cells, the western blot results revealed that BFP-TA inhibited the elevation of the protein levels of Bax, MST1, and FOXO3a in CSE-induced Beas-2B cells, suggesting that BFP-TA may attenuate CSE-induced apoptosis by inhibiting the expression of Bax, MST1 and FOXO3a. Furthermore, COPD is a common disease of respiratory system characterized by chronic inflammation of the lungs, airway inflammation is a key feature of COPD, and the MAPK signaling pathway is a classical pathway that regulates inflammatory signaling. Many studies have shown that cigarette smoke induces inflammatory responses by activating the expression of the MAPK signaling pathway (30–32). In this study, the western blot results showed that CSE induced a significant increase in the phosphorylation levels of JNK, ERK and P38, and the BFP-TA intervention could attenuate the effect of CSE, suggesting that BFP-TA could inhibit the activation of the MAPK signaling pathway.

*In vitro* cellular experiments showed that BFP-TA could attenuate CSE-induced oxidative damage and apoptosis, but the target genes are still unclear. Therefore, to further investigate the effect of BFP-TA on gene expression in CSE-induced Beas-2B cells, Illumina sequencing technology was used to perform transcriptome sequencing, bioinformatic analysis, screening of DEGs, systematic analysis of the relevant biological functions and possible mechanism to search for possible target genes for the treatment of COPD. Subsequently, KEGG enrichment analysis revealed multiple pathways associated with COPD, including p53 signaling pathway, MAPK signaling pathway, PI3K-Akt signaling pathway, Hippo signaling pathway, Nicotine addiction, PPAR signaling pathway, Cholesterol metabolism, etc., which was consistent with previous studies (33–38), and could initially confirm the reliability of this study. Among the top 20 signaling pathways enriched based on key DEGs, we are most interested in the PPAR signaling pathway and the PI3K-Akt signaling pathway.

PPAR signaling pathway is mainly associated with lipid and glucose metabolism, inflammation and cancer. Studies have shown that PPARγ is a nuclear hormone receptor that plays a key role in the inflammatory process of COPD (39). MMP1 is a member of the matrix metalloproteinase family of proteins, which are mainly involved in the breakdown of extracellular matrix, and the elevated expression of MMP1 is closely related to the pathogenesis of COPD (40, 41). ANGPTL4 encodes a glycosylated secretory protein that plays a role in the regulation of glucose homeostasis, lipid metabolism and insulin sensitivity. Studies have shown that the ANGPTL4 level of serum was significantly elevated in COPD patients (42), and the overexpression of ANGPTL4 was associated with lung function and inflammation (43). Many studies have shown that the remodeling of the lung’s lipidomics is observed in respiratory diseases such as asthma, COPD, and pneumonia (44). Disturbed pulmonary lipid metabolism is an important pathophysiological feature of the COPD process (38). The FADS2 gene mainly encodes proteins of the fatty acid desaturase family, and studies have shown that FADS2 level in serum was significantly decreased in LPS-induced acute inflammation mice (45). SCD encodes an enzyme involved in fatty acid biosynthesis, and studies have shown that inhibition of SCD1 will exacerbate cigarette smoke-induced inflammatory injury in mice (46). OLR1 encodes a low-density lipoprotein receptor (LOX-1) that is associated with vascular injury and inflammatory responses and studies have shown that LOX-1 could attenuate pneumonia-induced lung injury (47). In this study, the sequencing results showed that BFP-TA could downregulate the mRNA expression of MMP1 and ANGPTL4, while upregulated the mRNA expression of FADS2, SCD and OLR1 in CSE-induced Beas-2B cells, which was consistent with the qPCR results. These results suggest that BFP-TA may modulate CSE-induced inflammatory responses and lipid metabolism disorders by regulating the transcript levels of MMP1, ANGPTL4, FADS2, SCD and OLR1 in the PPAR signaling pathway.

Furthermore, the PI3K-Akt signaling pathway is a key regulatory pathway in the inflammatory and oxidative stress response in COPD and plays a critical role in disease progression and treatment (48). Studies have shown that CCND1 is a key mediator of respiratory remodeling (49, 50). EPHA2 is a tyrosine kinase receptor on the cell membrane and is a key regulator of inflammatory and injurious disorders (51). Studies have shown that EPHA2 antagonists could inhibit inflammatory factor release and inflammatory cell infiltration in BALF of rats and inhibit the elevation of MPO and MDA levels, thereby exerting a protective effect against LPS-induced acute lung injury (52). ITGB4 is a cell-surface protein involved in cell adhesion as well as cell-surface-mediated signaling, and studies have shown that the expression of ITGB4 in airway epithelial cells was significantly downregulated under oxidative stress or inflammatory stimuli (53, 54). DDIT4 is mainly involved in apoptosis and response to hypoxia, and its expression level was increased under the stresses of chemotherapy, hypoxia and DNA damage (55, 56). In this study, the sequencing results showed that BFP-TA could downregulate the expression of CCND1 and EPHA2, while upregulate the expression of ITGB4 and DDIT4 in CSE-induced Beas-2B cells, which was consistent with the qPCR results. These results suggest that BFP-TA may alleviate CSE-induced inflammation, oxidative stress and apoptosis by modulating the transcript levels of CCND1, EPHA2, ITGB4 and DDIT4 in the PI3K-Akt signaling pathway. Furthermore, although some important discoveries are revealed by this study, there are also some limitations. Firstly, the specific active components and pharmacokinetics studies of BFP-TA still need further investigation. Secondly, the specific molecular mechanism and signal transduction process of BFP-TA in anti-COPD still need further investigation. And we will consider these questions seriously in our next work.

## Conclusion

This is the first study to investigate the protective effect and molecular mechanism of BFP-TA in CSE-induced Beas-2B cell injury model. The results showed that BFP-TA could attenuate CSE-induced oxidative damage, apoptosis and inflammation in Beas-2B cells, and the mechanism may be related to the regulation of apoptosis-related proteins, SIRT1/Nrf2/Keap1 and MAPK signaling pathways. Subsequently, the effect of BFP-TA on CSE-induced gene expression in Beas-2B cells was further analyzed by transcriptome sequencing. The results showed that BFP-TA could exert anti-inflammatory, antioxidant, and anti-apoptotic effects and regulate lipid metabolism disorders by regulating the expression of DEGs in PPAR and PI3K-Akt signaling pathways, thus exerting a protective effect against CSE-induced Beas-2B cell injury. This study will provide data support for elucidating the protective effect and molecular mechanism of BFP-TA on Beas-2B cell injury model and developing new drugs for the treatment of COPD.

## Data availability statement

The data that support the findings of this study are available from the corresponding author upon reasonable request.

## Conflict of interest and funding

The authors declare that they do not have any conflict of interest. This research was supported by the major science and technology research project in 2021 from Tibet Science and Technology Department (NO. XZ202101ZD0021G), the science and technology major project of Tibetan Autonomous Region of China (NO. XZ202201ZD0001G01, NO. XZ202201ZD0001G06), the Funds for local scientific and technological development guided by the central government in 2023 from Tibet Science and Technology Department (NO. XZ202301YD0014C), the Major science and technology research project in 2023 from Tibet Science and Technology Department (NO. XZ202301ZY0009G), the Key support projects of the central government for local transfer payment funds (inheritance and development of traditional Chinese medicine) in the Xizang Autonomous Region in 2023 (NO. 2023-XZ-ZYYJ-01-LH).

## Authors’ contributions

Implementing the research process, formal analysis, writing-original draft: Xiaoyu Wang; Writing review and editing: Xiao Liu; writing review and editing: Er-Bu AGA; supervision, project administration: Wai Ming Tse; supervision, project administration: Kathy Wai Gaun Tse; writing review and editing, supervision, project administration, funding acquisition: Bengui Ye.

## Supplementary Material


