# Supplementary material for: Protective effect of the total alkaloid extract from Bulbus Fritillariae Pallidiflorae on cigarette smoke-induced Beas-2B cell injury model and transcriptomic analysis
Source: Food Nutr Res. 2024 Jun 11;68:10.29219/fnr.v68.10689. doi: 10.29219/fnr.v68.10689 (PMC11227262; doi:10.29219/fnr.v68.10689)
Supplement: Supplementary file 1 [file FNR-68-10689-s1.docx]

**Supplementary Material**

# Material and methods

***Preparation of BFP-TA and determination of total alkaloid content***

The appropriate amount of Bulbus *Fritillariae Pallidiflorae* (BFP) was pulverized into powder (over 24 mesh sieve), then reflux extracted twice with 70% ethanol (material-liquid ratio = 1:10), the first time for 4 hours and the second time for 3 hours. After filtering the dregs, the filtrate was combined and concentrated under reduced pressure to obtain the dry extract. The dry extract was dissolved in 2% HCl, then filtered again and adjusted to pH = 8 with NaOH solution, and the filtrate was used as an adsorbent. The prepared adsorbent was loaded on the H103 macroporous adsorbent resin and eluted with water and 30% ethanol sequentially until the eluate was negative for the molish reaction, then eluted with 90% ethanol and anhydrous ethanol solution until the eluate was negative for the alkaloid reaction. Subsequently, the 90% ethanol and anhydrous ethanol eluent were collected and concentrated to dryness under reduced pressure to obtain the total alkaloid extract from BFP (BFP-TA).

The content of total alkaloids was calculated with the method of "content determination" in the “*Fritillariae Cirrhosae* Bulbus” according to the Chinese Pharmacopoeia (2020 Edition). The content of total alkaloids was determined by UV-Vis spectrophotometry (Beijing Purkinje General Instrument Co., Beijing, China), using imperialine as the standard compound. The absorbance was measured at 415 nm, and the total alkaloid content was calculated based on the calibration equation obtained from imperialine.

***HPLC-ELSD and UHPLC-MS/MS analysis of BFP-TA***

HPLC-ELSD method: After the BFP-TA sample was dissolved in chromatographic methanol to 20 mg/mL, the solution was filtered through a 0.22 μm filter (Agilent, CA, USA) into an HPLC vial. The chromatographic separation was achieved on an Eclipse Plus C18 (250 mm × 4.6 mm, 5 μm), with a temperature stated at 25 °C. Analysis was completed with a gradient elution of 0.03% diethylamine (A) and acetonitrile (B), and the gradient program was as follows: 0–10 min, 30% B; 10–35 min, 30%→60% B; 35-45min, 60% B; 45-65min, 60%→90% B; 65-75min, 90% B; 75-80min, 90%→30% B; 80-90min, 30% B. The flow rate was 1.0 mL/min. The injection volume was 10 μL, with the SEDERE SEDEX 90 evaporative light scattering detector (Dikma, Beijing, China) at a gasification temperature of 40°C and air pressure of 3.5 bar**.**

UHPLC-MS/MS method: The chromatographic separation was achieved on an Agilent ZORBAX SB-C18 (100 mm × 2.1 mm, 1.8 μm), temperature stated at 40 °C. The analysis was completed with a gradient elution of 0.1% formic acid in purified water (A) and acetonitrile (B), and the gradient program was as follows: 0.0-0.5 min, 15%→15% B; 0.5-3.5 min, 15%→45% B; 3.5-4.5 min, 45%→50% B; 4.5-6.0 min, 50%→95% B; 6.0-8.0 min, 95%→95% B; 8.0-8.1 min, 95%→15% B; 8.1-11.0 min, 15%→15% B; 11.0 min, stop. The flow rate was 0.3 mL/min, and the injection volume was 1 μL.

The UHPLC system was coupled to the QQQ-MS/MS instrument (Shimadzu, Kyoto, Japan), operating in positive electrospray ionization (ESI) mode. The parameters for MS detection were as follows: the injection voltage was set to 5500 V, and the ion source temperature was set to 450 °C. The atomized gas (Gas1) was at 50.0 psi, the heated gas (Gas2) was at 50.0 psi, and the curtain gas (CUR) was at 35.0 psi. Multiple reaction monitoring (MRM) was used for scanning; the collision gas (CAD) pressure was 9.0, and the Q1 and Q3 resolutions were UNIT. Data acquisition and processing were performed using Analyst Software 1.7.0 and SCIEX OS 1.6.1 software. For the qualitative and quantitative analysis of BFP-TA, eight isosteroidal alkaloids were used as standards, namely edpetiline, imperialine, peimisine, yibeinoside A, verticinone, isopeimine, delavinone, and ebeiedinone. Then the content of the eight isosteroidal alkaloids in BFP-TA was calculated based on the calibration equation obtained from the standards.

**Results**

***Material basis research of BFP-TA***

The content of eight alkaloids is calculated according to the calibration equation within the linear range of each standard compound. The linear relationships and ranges of the eight standard compounds are shown in Table S1, and the mass spectrum detection data of eight alkaloids are shown in Table S2.

***Table S1.*** The linear relationships and ranges of the eight reference components

| Identification compound | calibration equation | R^2^ | linear ranges（ng∙mL^-1^) |
| --- | --- | --- | --- |
| Edpetiline | Y=2.169E4 X +4.959E6 | 0.9938 | 125.000~2000.000 |
| Imperialine | Y=4.727E4 X +6.492E6 | 0.9928 | 125.000~2000.000 |
| Peimisine | Y=1.200E4 X +2.732E4 | 0.9965 | 3.125~50.000 |
| Yibeinoside A | Y=8.160E3 X +1.398E5 | 0.9968 | 18.750~300.000 |
| Verticinone | Y=2.534E4 X +9.545E4 | 0.9982 | 3.125~50.000 |
| Isopeimine | Y=2.030E5 X +6.424E5 | 0.9971 | 3.125~50.000 |
| Delavinone | Y=6.046E3 X +8.155E5 | 0.9978 | 125.000~2000.000 |
| Ebeiedinone | Y=1.267E4 X +5.027E5 | 0.9968 | 50.000~800.000 |

***Table S2.*** The mass spectrum detection data of eight alkaloids

| Identification compound | Retention Time (min) | Content | Q1 Mass  (Da) | Q3 Mass  (Da) | DP  (Volts) | CE  (Volts) |
| --- | --- | --- | --- | --- | --- | --- |
| Edpetiline | 2.987 | 13.29% | 592.4 | 574.4 | 57 | 40 |
| Imperialine | 3.493 | 34.68% | 430.4 | 138.1 | 60 | 100 |
| Peimisine | 3.554 | 0.22% | 428.3 | 114.1 | 40 | 230 |
| Yibeinoside A | 3.622 | 1.18% | 576.4 | 414.4 | 80 | 150 |
| Verticinone | 3.876 | 0.80% | 430.3 | 148.2 | 80 | 180 |
| Isopeimine | 4.137 | 0.24% | 432.4 | 414.4 | 47 | 40 |
| Delavinone | 4.255 | 6.92% | 414.4 | 98.1 | 65 | 40 |
| Ebeiedinone | 4.527 | 3.28% | 414.4 | 91.1 | 110 | 130 |

***Transcriptome Analysis***

***Table S3.*** DEGs in the Top 20 signaling pathways of the KEGG enrichment analysis

| Term | Classification | Gene ID |
| --- | --- | --- |
| p53 signaling pathway | CellP. | CCND1; CCNE2; TNFRSF10A; TP73 |
| Focal adhesion | CellP. | CCND1; COL4A4; ITGB4; ITGB8; PDGFB; PGF; THBS2 |
| Viral protein interaction with cytokine and cytokine receptor | EnvIP. | ACKR3; CCL20; TNFRSF10A; TNFRSF10D; TNFSF10; TNFSF14 |
| MAPK signaling pathway | EnvIP. | AREG; CACNA2D4; DUSP5; DUSP6; EPHA2; FOS; HSPA6; IGF2; KIT; MAP3K8; NGFR; PDGFB; PGF; RASGRP3 |
| PI3K-Akt signaling pathway | EnvIP. | AREG; CCND1; CCNE2; COL4A4; DDIT4; EPHA2; IGF2; ITGB4; ITGB8; KIT; MYB; NGFR; PDGFB; PGF; PPP2R2B; THBS2 |
| Ras signaling pathway | EnvIP. | EPHA2; GRIN2A; HTR7; IGF2; KIT; NGFR; PDGFB; PGF; PLD1; RASGRP3 |
| Cytokine-cytokine receptor interaction | EnvIP. | ACKR3; BMP8B; CCL20; IL11; NGFR; TNFRSF10A; TNFRSF10D; TNFRSF13C; TNFRSF19; TNFRSF9; TNFSF10; TNFSF14 |
| Hippo signaling pathway | EnvIP. | AREG; BMP8B; CCND1; PPP2R2B; TP73; WNT2B |
| Nicotine addiction | HumaD. | CHRNB2; GABRQ; GRIN2A |
| Bladder cancer | HumaD. | CCND1; E2F2; MMP1 |
| Pancreatic cancer | HumaD. | ARHGEF6; CCND1; E2F2; PLD1 |
| Prostate cancer | HumaD. | CCND1; CCNE2; E2F2; ETV5; PDGFB |
| Measles | HumaD. | CCND1; CCNE2; FOS; HSPA6; MX2; OAS2; TP73 |
| MicroRNAs in cancer | HumaD. | BMF; CCND1; CCNE2; DDIT4; E2F2; HMOX1; PDGFB |
| Cushing syndrome | HumaD. | CCND1; CCNE2; E2F2; PBX1; PDE8B; WNT2B |
| Transcriptional misregulation in cancer | HumaD. | DUSP6; ETV1; ETV4; ETV5; ETV7; NGFR; PBX1 |
| Human papillomavirus infection | HumaD. | CCND1; CCNE2; COL4A4; HES4; ITGB4; ITGB8; MX2; PPP2R2B; THBS2; WNT2B |
| Pathways in cancer | HumaD. | CCND1; CCNE2; COL4A4; E2F2; FOS; HMOX1; IGF2; KIT; MMP1; PDGFB; PGF; PLD1; RASGRP3; WNT2B |
| PPAR signaling pathway | OrgaS. | ANGPTL4; FADS2; LPL; MMP1; OLR1; SCD |
| Cholesterol metabolism | OrgaS. | FANGPTL4; LPL; PCSK9; SOAT2 |

***Table S4.*** The expression levels of validated DEGs

| Gene name | log_2_ (FC)  (CSE/C) | log_2_(FC)  (BFP/CSE) | FPKM(C) | FPKM(CSE) | FPKM(BFP) |
| --- | --- | --- | --- | --- | --- |
| MMP1 | 5.01 | -1.63 | 0.60±0.21 | 19.68±0.59 | 6.45±0.27 |
| ANGPTL4 | 4.61 | -2.57 | 1.00±0.2 | 25.01±1.85 | 4.27±0.39 |
| CCND1 | 2.28 | -1.45 | 7.00±0.16 | 34.69±1.22 | 12.92±0.37 |
| EPHA2 | 1.10 | -1.17 | 27.24±1.02 | 59.58±1.31 | 26.99±0.72 |
| FADS2 | -1.12 | 1.56 | 69.66±0.88 | 32.67±1.06 | 97.72±0.74 |
| DDIT4 | -1.25 | 1.11 | 21.04±1.69 | 9.01±0.3 | 19.84±0.82 |
| SCD | -1.53 | 2.26 | 35.68±1.81 | 12.55±1.1 | 61.00±0.54 |
| OLR1 | -2.12 | 1.34 | 12.67±0.76 | 2.96±0.18 | 7.61±0.32 |
| ITGB4 | -2.51 | 1.55 | 5.12±0.015 | 0.90±0.029 | 2.63±0.029 |
